# Hebbian learning in the MSO: emergence of interaural tuning

**DOI:** 10.1186/1471-2202-15-S1-P198

**Published:** 2014-07-21

**Authors:** P Yger, V Benichoux, M Stimberg, R Brette

**Affiliations:** 1 Institut d’Etudes de la Cognition, Ecole Normale Supérieure, Paris, France; 2 Sorbonne Universités, UPMC Univ. Paris 06, UMR S 968, Institut de la Vision, Paris, F-75012, France; 3 INSERM, U968, Paris, F-75012, France; 4 CNRS, UMR 7210, Paris, F-75012, France

## 

To localize sounds in the environment, animals mostly use spectro-temporal cues originating from the physical disparities of the sound waveforms impacting the ears. Among those, the Interaural Time Difference (ITD) has been shown to be crucial in mammals for locating low-frequency sounds, and is known to be processed by neurons in a particular structure, the Medial Superior Olive (MSO). While it is classically considered that the emergence of ITD selectivity in a neuron of the MSO is due to differences in the axonal delays originating from the two ears and impinging the cell (the so called “delay-line” model [1]), experimental evidence shows that the best delay (the ITD at which the neuron’s firing rate is maximum) is also dependent on the frequency of the sound [2].

Because such an observation is a challenge to the classical model, we want to investigate the emergence of binaural tuning in the MSO through Hebbian learning, similarly to what has already been done in [3]. To do so, we built a realistic neuronal architecture based on spiking neurons and using homeostatic and spike-timing dependent plasticity rules for synapses from cochlear nucleus projections to neurons in the MSO. By training the system with binaural sounds, we were able to study the development of ITD selectivity for various inputs and to understand why, and how, this ITD selectivity can depend on the frequency of the sound.

Finally, we will discuss, from a coding point of view, the potential implications raised by the frequency dependence of the best delay. As pointed out by recent work [4], with such a frequency-dependent best delay, neurons in the MSO should be seen as coding for a particular position in space, rather than for just a fixed delay difference.

## References

[B1] Jeffress LA A place theory of sound localization J. Comp. Physiol. Psychol 1948 15 35 39 18904764 10.1037/h0061495

[B2] Joris P Yin TC A matter of time: internal delays in binaural processing Trends Neurosci 2007 15 2 70 8 10.1016/j.tins.2006.12.004 17188761

[B3] Fontaine B Brette R Neural Development of Binaural Tuning through Hebbian Learning Predicts Frequency-Dependent Best Delays J. Neurosci 2011 15 32 11692 11696 10.1523/JNEUROSCI.0237-11.2011 21832198 PMC6623129

[B4] Benichoux V Fontaine B Karino S Joris P Brette R Frequency-dependent time differences between the ears are matched in neural tuning (submitted) 10.7554/eLife.06072 PMC4439524 25915620

